# High-Throughput Sequencing to Investigate lncRNA-circRNA-miRNA-mRNA Networks Underlying the Effects of Beta-Amyloid Peptide and Senescence on Astrocytes

**DOI:** 10.3389/fgene.2022.868856

**Published:** 2022-05-12

**Authors:** Yuxin Deng, Hui Song, Yan Xiao, Yi Zhao, Liangzao Chu, Jiuyang Ding, Xiangchun Shen, Xiaolan Qi

**Affiliations:** ^1^ Key Laboratory of Endemic and Ethnic Diseases, Ministry of Education and Key Laboratory of Medical Molecular Biology of Guizhou Province, Guizhou Medical University, Guiyang, China; ^2^ School of Basic Medical Sciences, Guizhou Medical University, Guiyang, China; ^3^ Department of Neurosurgery, Affiliated Hospital of Guizhou Medical University, Guiyang, China; ^4^ School of Forensic Medicine, Guizhou Medical University, Guiyang, China; ^5^ School of Pharmaceutical Sciences, Guizhou Medical University, Guiyang, China; ^6^ Translational Medicine Research Center, Guizhou Medical University, Guiyang, China

**Keywords:** ceRNA, alzheimer’s disease, amyloid beta, senescence, focal adhesion

## Abstract

Astrocytes are widely distributed in the central nervous system and play an essential role in the function of neuronal cells. Associations between astrocytes and Alzheimer’s disease (AD) have been noted, and recent work has implicated circular RNA (circRNA) and long non-coding RNA (lncRNA) in the development of AD. However, few reports have investigated which lncRNA and circRNA are involved in the influence of amyloid beta (Aβ) and senescence on astrocytes. This study therefore examines changes at the transcriptome level to explore the effects of Aβ and senescence on astrocytes. Primary cultured astrocytes were treated with Aβ and cultured for 90 days *in vitro*, and high-throughput sequencing was performed to identify differentially expressed RNAs. Gene Ontology and Kyoto Encyclopedia of Genes and Genomes enrichment analyses revealed that differentially expressed genes were associated with the focal adhesion signaling pathway, extracellular matrix receptor signaling pathway, and the extracellular matrix. The protein–protein interaction network was then constructed, and 103 hub genes were screened out; most of these were strongly associated with the expression of the extracellular matrix, extracellular matrix receptor signaling pathway, and focal adhesion. Two competing endogenous RNA networks were constructed based on the selected hub gene and differential RNAs, and we identified multiple competing endogenous RNA regulatory axes that were involved in the effects of Aβ and senescence on astrocytes. This is the first study to explore the molecular regulation mechanism of Aβ and senescence on primary astrocytes from the perspective of the whole transcriptome. In uncovering the signaling pathways and biological processes involved in the effects of Aβ and senescence on astrocytes, this work provides novel insights into the pathogenesis of AD at the level of competing endogenous RNA network regulation.

## Introduction

Amyloid beta-peptide (Aβ) accumulation is a predominant pathological feature of Alzheimer’s disease (AD) (Ege and Lee, 2004), which is the most common form of age-related dementia worldwide (Dunys et al., 2014). With the aging population, the morbidity of AD has been steadily rising, posing a serious threat to human health. However, the available drug treatment options are limited in their efficacy (Wilkins and Swerdlow, 2017; Xu et al., 2020). Typically, work in the field of AD has focused on the direct toxic effect of Aβ on neurons; however, recent studies have shifted the paradigm to reveal the role of astrocytes in neurodegenerative diseases.

Astrocytes, the most abundant type of glial cells in brain tissues, have recently received significant attention due to their unique neuron-safeguarding functions. Astrocytes are typically star-shaped glial cells that are closely associated with neuronal nutritional support, blood–brain barrier formation, extracellular ion homeostasis, and neurosynaptic remodeling (Xian et al., 2019). Reactive astrogliosis surrounding amyloid plaque is a neuropathological hallmark of AD (Xu et al., 2015). Although the number of studies on astrocyte senescence has increased, the exact mechanism underlying the role of senescent astrocytes in AD progression is still not completely understood.

Non-coding RNAs (ncRNAs), which include long non-coding RNAs (lncRNAs) and circular RNAs (circRNAs), are RNA molecules discovered in recent decades. ncRNAs are widely considered as microRNA sponges that do not have the function of being translated into proteins. lncRNAs are characterized by a length of more than 200 bases (Derrien et al., 2012), and circRNAs have recently been identified as ncRNAs with covalent closed structures that regulate disease development and occurrence (He et al., 2017; Zhong et al., 2018; Wu et al., 2020). circRNAs are hundreds to thousands of bases long and are formed by shearing and cyclization of introns or exons and have been shown to act as a microRNA (miRNA) sponge (Hansen et al., 2013). Both lncRNAs and circRNAs regulate subsequent transcription and translation processes by competitively binding to the metal response element of the corresponding miRNA. Nevertheless, there have been few reports on RNA-mediated regulatory networks of astrocytes in AD pathogenesis, and there is not yet an established understanding of RNA-mediated regulatory networks. A systematic study of the molecular mechanisms of astrocyte-associated RNAs in AD pathogenesis is therefore essential to identify novel targets for Aβ toxicity of astrocytes in AD.

To investigate the role of ncRNAs in the effects of Aβ and senescence on astrocytes, we investigated the differential expression of lncRNAs (DElncRNAs), circRNAs (DEcircRNAs), miRNAs (DEmiRNAs), and messenger RNAs (DEmRNAs) in each group of astrocytes using high-throughput sequencing. Differentially expressed RNAs were identified by differential expression analysis, and based on ceRNA regulatory principles, the competitive endogenous RNA (ceRNA) regulatory network and protein–protein interaction (PPI) network involved in the effects of Aβ and senescence on astrocytes were constructed. Gene Ontology (GO) and Kyoto Encyclopedia of Genes and Genomes (KEGG) enrichment analyses were then performed with the differentially expressed genes (DEGs) identified. To our knowledge, this is the first time that ceRNA networks established by circRNA and lncRNA have been used to explore the molecular mechanisms underlying Aβ and senescence interactions with astrocytes.

## Materials and Methods

### Ethics

This study was conducted in accordance with ethical standards of national and international guidelines and was approved by the Ethics Committee of Guizhou Medical University.

### Preparation of the Aβ_42_ Oligomer Solution

The Aβ_42_ oligomer solution was prepared in accordance with our previously reported method (Wang et al., 2012). Synthetic Aβ_42_ was suspended in prechilled hexafluoroisopropanol at a final concentration of 1 mM and then incubated for 60 min at room temperature, followed by 10 min on ice. Aliquots of the Aβ_42_ solution were transferred to non-silicified microcentrifuge tubes, and hexafluoroisopropanol was left to evaporate overnight in the hood at room temperature. The tubes were then stored at −80°C. Before treatment of the cells, Aβ_42_ was dissolved in dimethyl sulfoxide to obtain a final concentration of 5 mmol/L. To prepare the oligomers, the solution was diluted to a modified Dulbecco’s modified Eagle’s medium dilution and then incubated at 4°C for 24 h.

### Cell Culture and Treatment

Primary cultured astrocytes were cultured and identified as previously described (Galland et al., 2019). Briefly, neonatal Sprague–Dawley rats were sterilized in 75% alcohol and sacrificed, and their bilateral cortices were collected. The tissue was cut into 1 mm^3^ cubes and digested with 0.25% trypsin for 11 min at 37°C. The digestion was terminated with Dulbecco’s modified Eagle’s medium (11965092, Gibco/Thermofisher, China) containing 10% fetal bovine serum and then gently pipetted 10–15 times to produce single-cell suspensions. These were then filtered through a 40 μm filter sieve, diluted to the appropriate cell concentration, and added to the polylysine-coated cell culture flasks for culture. The medium used was Dulbecco’s modified Eagle’s medium/F12 medium (c11330500BT, Gibco/Thermofisher, China) containing 10% fetal bovine serum, 100 units of penicillin, and streptomycin. Once the growth of astrocytes had been stabilized and the cell confluence reached 90% or more, the astrocytes were purified using a thermostatic shaker and identified by immunofluorescence using glial fibrillary acidic protein (1:1000) (ab7260, Abcam, Cambridge, United Kingdom) as a biomarker. Each experiment was performed with at least five samples, and each determination was made in triplicate unless indicated otherwise. The primary cultured cells were divided into three groups. The control group was routinely cultured for 12 days (group x12), the natural aging group was routinely cultured for 90 days (group x90), and the Aβ_42_ treatment group was cultured for 12 days and then treated with a medium containing 10 μmol/L Aβ_42_ oligomers for 48 h (group x12A).

### Senescence-Associated β-Galactosidase Activity Assays

Senescence-associated β-galactosidase (SA-β-gal) is a lysosomal enzyme that becomes more active when aging is initiated (Sikora et al., 2021). In the present study, SA-β-Gal activity was used to detect the onset of senescence in primary cells. After being cultured and purified, primary astrocytes were cultured *in vitro* for 90 days and treated with 10 μM Aβ_42_ for 48 h, and senescent cells were stained using the SA-β-Gal staining kit (BC2580, Solarbio, Beijing, China). Briefly, the cells were fixed with the fixation buffer and incubated with an X-gal-containing staining solution at 37°C overnight. Microscopic images were taken for positive cell counting and statistical analysis. Integrated optical density was used to evaluate the relative changes in β-galactosidase activity.

### Whole Transcriptome RNA Library Preparation and Sequencing

Library construction and RNA sequencing were performed by Novogene Co., Ltd. (Beijing, China). The total RNA was extracted from each sample using the TRIzol reagent (Invitrogen, United States), and the concentration and purity of the extracted RNA were evaluated using a NanoPhotometer® spectrophotometer. The lncRNA/mRNA and circRNA libraries were constructed by removing ribosomal RNA using an NEBNext rRNA depletion kit v2 (NEB,#E7400) and NEBNext Ultra™ II RNA library prep kit for Illumina (NEB, #E7760). First, the ribosomal RNA was removed from the total RNA, followed by fragmentation of the RNA into short fragments of 250–300 bp using NEBNext First Strand Synthesis Reaction Buffer, the synthesis of the first-strand cDNA using the fragmented RNA as a template and random oligonucleotides as primers, and the synthesis of the second-strand cDNA using dNTPs (dUTP, dATP, dGTP, and dCTP) under the DNA polymerase I system. The purified double-stranded cDNAs are end-repaired, A-tailed, and connected to sequencing adapters, and the cDNAs of 350–400 bp were screened by AMPure XP beads. The second-strand cDNAs containing U are degraded by the USER enzyme, and finally, PCR amplification was performed to obtain libraries. By fragment length selection mentioned above, all RNAs except ribosomal RNA and small fragment RNAs (microRNA, siRNA, etc.) were finally obtained, including lncRNA, mRNA, and circRNA. After library preparation and pooling of different samples, the samples were subjected to Illumina sequencing (illumina Novaseq 6000). The lncRNA-seq used PE150 (paired-end 150 nt) sequencing for raw data. A total of 1 μg RNA per sample was used as an input material for the RNA sample preparations. For miRNA sequencing libraries, they were created separately, and after the samples had been tested and qualified, the libraries were constructed using the Small RNA Sample Pre Kit; this kit utilizes the special structure of the 3' and 5' ends of small RNA using total RNA as the starting sample and directly adds the adaptor to both ends of small RNA to synthesize cDNA. PCR products were purified on an 8% polyacrylamide gel (100 V, 80 min). DNA fragments corresponding to 140–160 bp (the length of small non-coding RNA plus the 3' and 5' adaptors) were recovered and dissolved in an 8 μL elution buffer. At last, library quality was assessed on an Agilent Bioanalyzer 2100 system using DNA high-sensitivity chips. The miRNA library preparations were sequenced on an Illumina Hiseq 2500/2000 platform, and 50 bp single-end reads were generated. A total of 1 μg total RNA per sample was used as an input material for the small RNA library. After library construction, Illumina sequencing was performed. Library construction and RNA sequencing were performed by Novogene Co., Ltd. (Beijing, China).

### Quality Control of Raw Sequencing Data

After the raw sequencing data had been acquired, internal Perl scripts were used to process and obtain clean reads without 5′ adapters, reads without 3′ adapters and with inserted sequences, and reads with unqualified quality. The Q20, Q30, and GC contents of the clean reads were calculated, and the clean reads of acceptable quality were used for subsequent calculation and analysis.

### Differential Expression Analysis

After performing quality control on the sequencing data, differential analysis was performed for x12A vs x12 groups and for the x90 vs x12 groups, and the intersection of DEGs was taken for x12A vs x12 and x90 vs x12 screening. Transcript and gene quantification was performed using StringTie software, and reads per kilobase of transcript per million mapped reads were obtained for quantification and differential expression analysis. Differential expression analysis was performed using Cuffdiff and edgeR software, and *p-*values were adjusted using Benjamini and Hochberg methods to control for false discovery rates. Genes with log2 (fold change) > 1.5 and padj < 0.05 were identified as DEGs for subsequent analysis.

### Gene Ontology and Kyoto Encyclopedia of Genes and Genomes Enrichment Analyses of Differentially Expressed Genes

The GO and KEGG enrichment analyses of DEGs were performed using the ClusterProfiler R package, and the gene length bias was corrected. Enrichment items with padj < 0.05 were defined as significant.

GO (Smith et al., 2003) analysis is an important enrichment analysis method in bioinformatics that enables the analysis of genes and proteins in three dimensions—cellular composition, molecular function, and biological processes. The KEGG (Kanehisa and Goto, 2000) signaling pathway database is a large database of genes, proteins, RNA, compounds, biochemical reactions, diseases, and other biological factors that documents the biological processes involved in each factor and facilitates predictive studies of their functions.

### Construction of Protein–Protein Interaction Networks

After screening for DEGs, the gene list was imported into STRING (https://string-db.org/) to construct a PPI network, and the network output was visualized by Cytoscape 3.7.0. Then, the Cytohubba (https://apps.cytoscape.org/apps/cytohubba) app was used to perform calculations on this network to filter hub genes, with degree as the filtering condition and the top 10% of the ranked genes as hub genes.

### Construction of the ceRNA Regulatory Network

In this study, a ceRNA network containing DEncRNA and DEmRNA was constructed to reveal potential regulatory relationships between lncRNAs, circRNAs, miRNAs, and mRNAs associated with Aβ and senescence. The miRanda (http://www.microrna.org/microrna) and TargetScan (http://www.targetscan.org/mmu_80) databases were used to predict miRNA binding seed sequence sites, and the prediction result network was visualized using Cytoscape 3.9.0 to show lncRNA-miRNA, circRNA-miRNA, and miRNA-mRNA inter-regulatory relationships.

### Validation of Hub Genes in the Alzdata Database

The hub genes screened by the PPI network and ceRNA network were input into the Alzdata database (http://www.alzdata.org/) for cross-checking. The hub gene lists were submitted for the normalized, differential, and convergent functional genomic rank analysis, compared with existing high-throughput sequencing data in the database, and visualized, and the tabulated results were output.

### Quantitative Real-Time PCR

To verify the reliability of transcriptome sequencing, the total RNA extracted from primary astrocytes of the x12, x12A, and x90 groups was used for quantitative real-time PCR (qRT-PCR) to detect expression level changes of selected genes. qRT-PCR was performed with the Bio-RAD CFX96 Real-Time PCR detection system using the RealStar Green Fast Mixture kit (GenStar, Beijing, China) according to the manufacturer’s instructions. Genes were normalized to RPS18 (Sangon Biotech, Shanghai, China). The primers used for qRT-PCR are listed in Table 1. The output Ct values were used for statistical analysis and were calculated as described previously (Chen et al., 2011):

1) ΔCt (Target gene Ct−averaged endogenous control Ct)
2) ΔΔCt (ΔCt_(sample)_− ΔCt_(Control group)_)
3) Fold change (2^−ΔΔCt^)

The t-test was used for statistical analysis, and the results are presented as the mean ± SD; n = 3, *p < 0.05, **p < 0.01, and ***p < 0.001.

**TABLE 1:** Primers for RT-qPCR.

Gene	Primer sequence (5′-3′)	Size (bp)
Ywhaz	F: ACT​ACT​ACC​GCT​ACT​TGG​CTG​AGG	83
	R: TTC​TTG​GTA​TGC​TTG​CTG​TGA​CTG​G	
Hdac1	F: ATG​AAG​CCT​CAC​CGA​ATC​CGA​ATG	111
	R: CTT​GGT​CAT​CTC​CTC​AGC​GTT​GG	
Epha4	F: GGA​AGG​AGG​GTG​GGA​GGA​AGT​AAG	113
	R: AGT​CAG​TTC​GCA​GCC​AGT​TGT​TC	
Anxa5	F: GAA​ACC​ATT​GAC​CGA​GAG​ACC​TCA​G	128
	R: TCC​GTC​CCA​GCA​CCC​TTC​ATA​G	
Bag3	F: AGG​AGA​GAT​GGC​GTC​AGG​AAG​G	109
	R: CAA​GGT​TGC​TGG​GCT​GGA​GTT​C	
Actb	F: CAC​TAT​CGG​CAA​TGA​GCG​GTT​CC	150
	R: ACT​GTG​TTG​GCA​TAG​AGG​TCT​TTA​CG	
Dnajc6	F: CCC​TCA​GAA​CCG​ACC​CAA​TTA​CAA​C	144
	R: GCA​TTG​AAG​CCT​TGA​CTG​GAA​AGC	

## Results

### Detection of Aβ_42_-Induced Senescence of Astrocytes In Vitro

**FIGURE 1:**
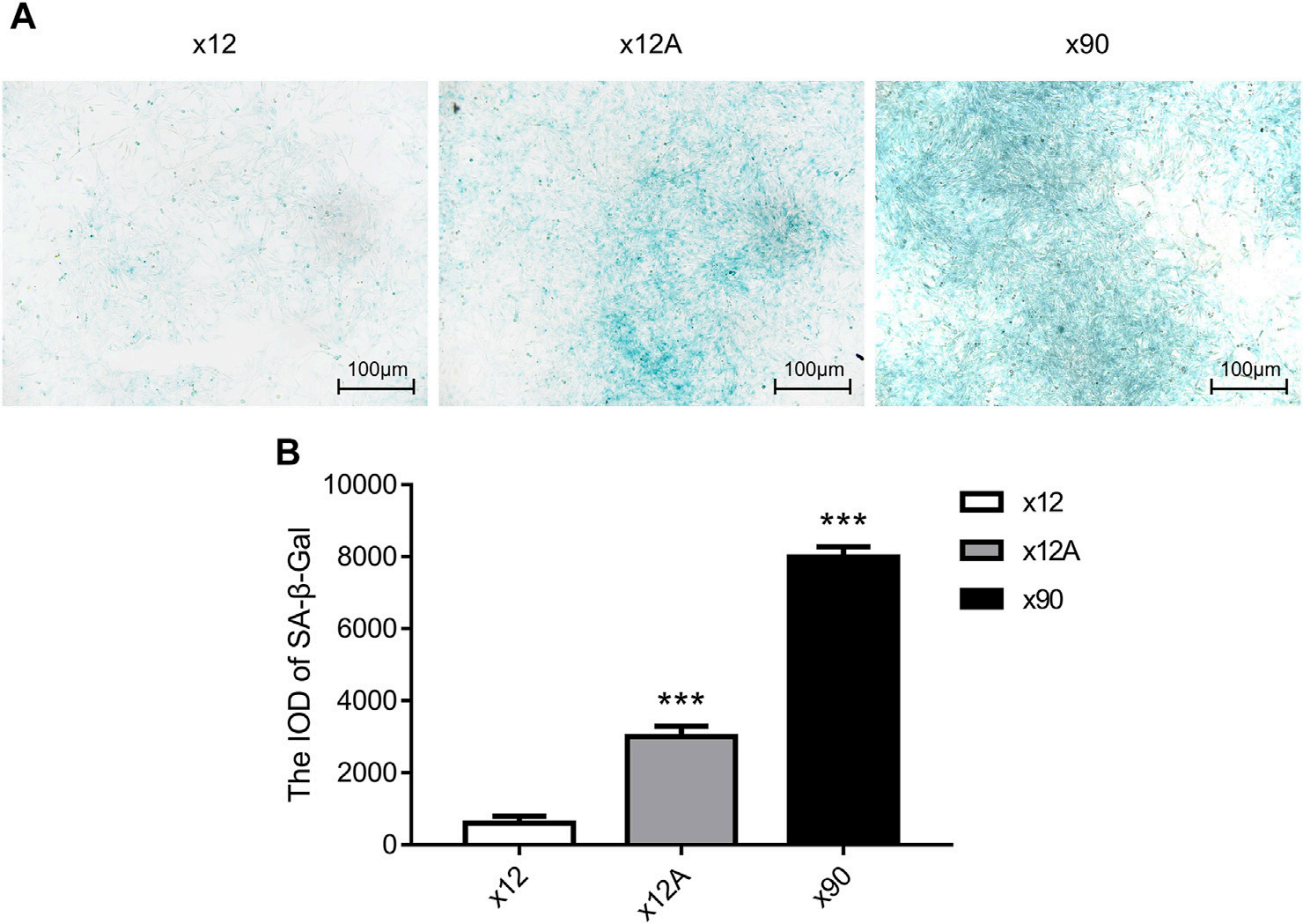
SA-β-gal staining of primary cultured astrocytes treated by Aβ and cultured for 90 days. **(A)** Representative images of SA-β-gal staining of primary cultured astrocytes treated with Aβ and cultured for 90 days. Green-stained astrocytes showed higher β-galactosidase activity. **(B)** Quantitative analysis of SA-β-gal-positive astrocytes. Scale bar = 100 μm. Data are expressed as the mean ± SEM. n = 5. **p < 0.01; ***p < 0.001 vs x12 group.

Senescent astrocytes were detected by SA-β-Gal staining after 90 days of *in vitro* culture and 48 h of treatment of primary astrocytes cultured *in vitro* with Aβ_42_. The results showed a significant increase in the number of positive SA-β-Gal staining cells in astrocytes exposed to Aβ_42_ and in senescent astrocytes, with more positive staining in astrocytes cultured *in vitro* for 90 days (*p* < 0.01; Figure 1).

### Overview of the Transcriptome Profiling

After the sequencing was completed, the obtained data were subjected to quality control to ensure the accuracy of the results and the RINs values were shown in Table 2. In the lncRNA/mRNA and circRNA libraries, 104039222, 105164930, and 104021862 clean reads were generated in the three samples of group x12, respectively; then, 99679302, 100587200, and 99018288 clean reads were generated in the three samples of group x12A, respectively. 105563836, 106589974, and 101629616 clean reads were generated in the three samples of group x90, respectively. In the miRNA library, 13617450, 16248447, and 14548621 clean reads were generated in the three samples of group x12; then, 14341829, 15264124, and 16557378 clean reads were generated in the three samples of group x12A, respectively, and in the three samples of group x90, respectively. 11685531, 14176739, and 10966249 clean reads were generated in the three samples of group x90. Detailed QC results are listed in Supplementary Table S5.

**TABLE 2:** Values of RINs for the samples

Sample	RINs
x12_1	9.1
x12_2	9.3
x12_3	9.4
x12A_1	8.7
x12A_2	8.9
x12A_3	8.8
x90_1	9.7
x90_2	9.4
x90_3	9.0

### Differential Expression Analysis

**FIGURE 2:**
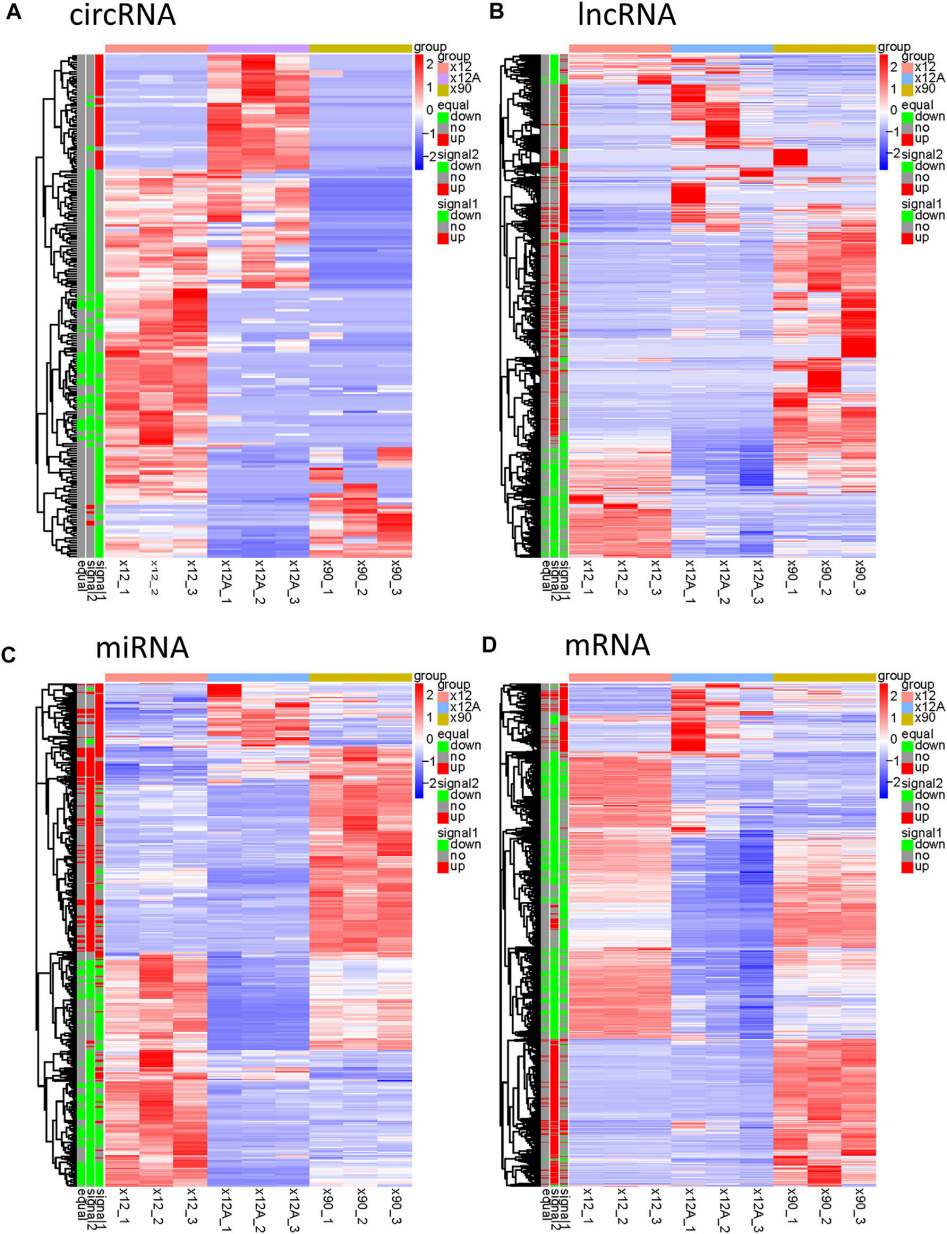
Hierarchical clustering and heat map analysis of differentially expressed RNAs. **(A)** circRNAs, **(B)** lncRNAs, **(C)** miRNAs, and **(D)** mRNAs in Aβ-treated and 90 day-cultured astrocytes *in vitro.* circRNA, circular RNA; lncRNA, long non-coding RNA; miRNA, microRNA; mRNA, messenger RNA. In the left legend, equal: genes that changed in both x12 and x90 groups together, signal1: genes that changed in the x12 group, signal2: genes that changed in the x90 group, red indicates upregulation, green indicates downregulation, and gray indicates insignificant change. The data were based on the predefined thresholds (padj<0.05, |log2FC|>1.5).

**FIGURE 3:**
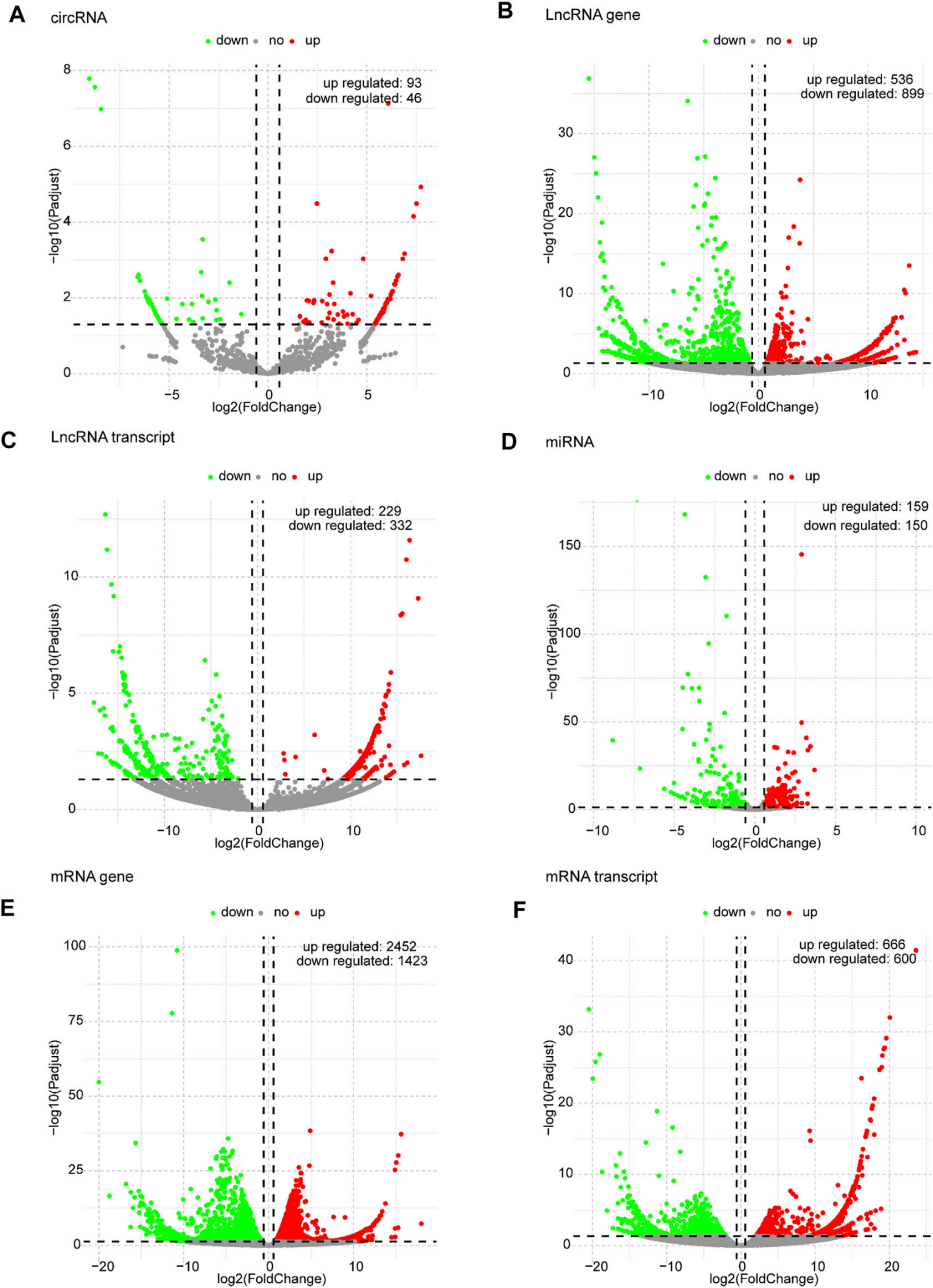
Volcano plots of differentially expressed RNAs in x12A astrocytes. **(A)** circRNA, **(B)** lncRNA gene level, **(C)** lncRNA transcript level, **(D)** miRNA, **(E)** mRNA gene level, and **(F)** mRNA transcript level in astrocytes treated with Aβ. Red and green indicate up and downregulation, respectively.

**FIGURE 4:**
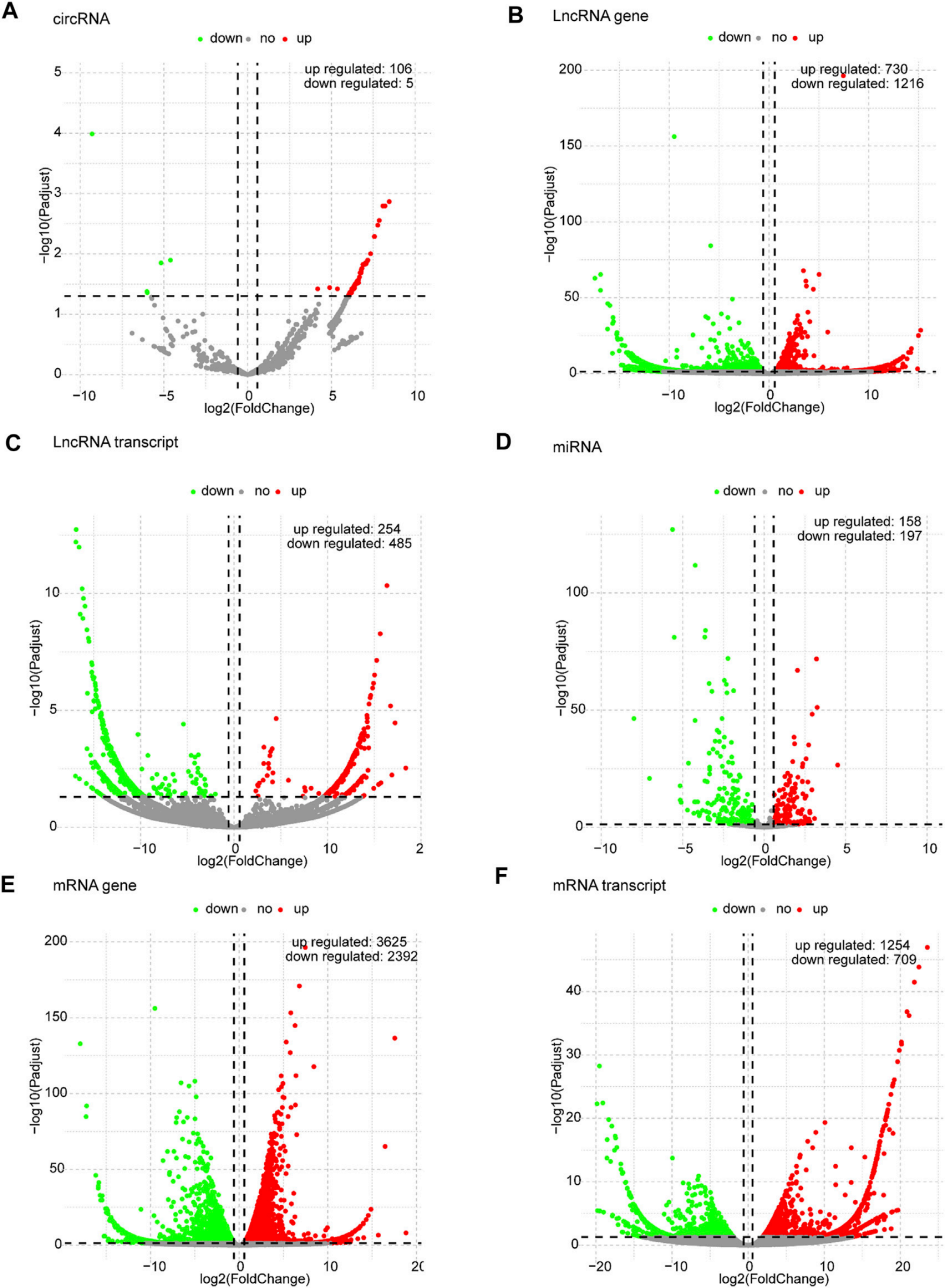
Volcano plots of differentially expressed RNAs in x90 astrocytes. **(A)** circRNA, **(B)** lncRNA gene level, **(C)** lncRNA transcript level, **(D)** miRNA, **(E)** mRNA gene level, and **(F)** mRNA transcript level in astrocytes cultured for 90 days *in vitro.* Red and green indicate up and downregulation, respectively.

**FIGURE 5:**
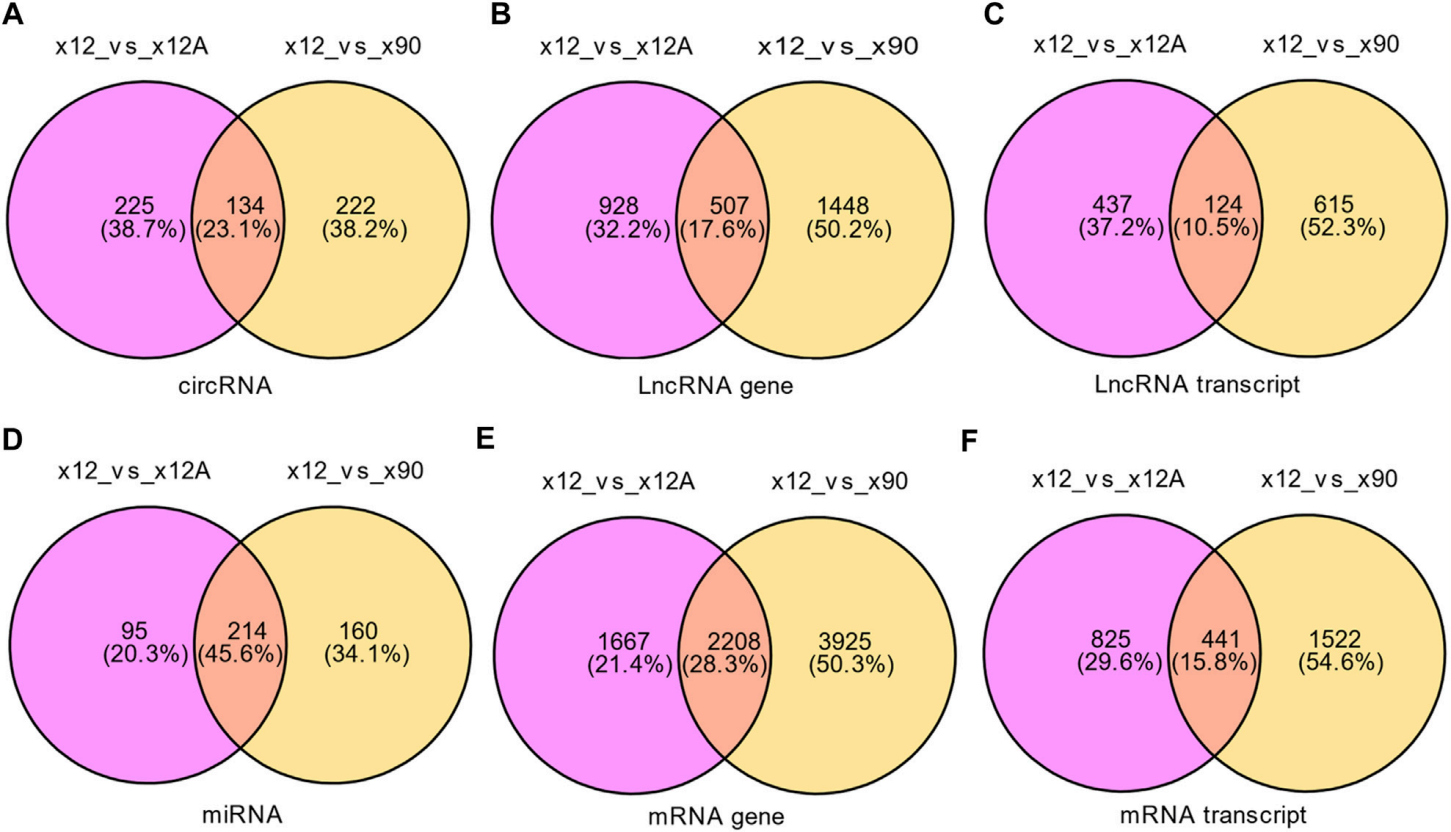
Venn diagram of differentially expressed RNAs in x12A and x90 astrocytes. **(A)** circRNA, **(B)** lncRNA gene level, **(C)** lncRNA transcript level, **(D)** miRNA, **(E)** mRNA gene level, and **(F)** mRNA transcript level in astrocytes treated with Aβ42 oligomers and cultured for 90 days *in vitro.* The data were based on the predefined thresholds (padj<0.05, |log2FC|>1.5).

Based on the predefined thresholds (padj<0.05, |log2FC|>1.5), the results showed remarkably different expression profiles of lncRNAs, circRNAs, miRNAs, and mRNAs between the three groups by the heat map (Figures 2A–D) and volcano map (Figures 3, 4). In the present study, 139 circRNAs (93 upregulated and 46 downregulated), 1435 lncRNAs (536 upregulated and 899 downregulated) at the gene level, 561 lncRNAs (229 upregulated and 332 downregulated) at the transcriptional level, 309 miRNAs (159 upregulated and 150 downregulated), 3875 mRNAs (2452 upregulated and 1423 downregulated) at the gene level, and 1266 mRNAs (666 upregulated and 600 downregulated) at the transcriptional level were identified as remarkably differentially expressed in the x12A group; a total of 111 circRNAs (106 upregulated and five downregulated), 1946 lncRNAs (730 upregulated and 1216 downregulated) at the gene level, 739 lncRNAs (254 upregulated and 485 downregulated) at the transcriptional level, 355 miRNAs (158 upregulated and 197 downregulated), 6017 mRNAs (3625 upregulated and 2392 downregulated) at the gene level, and 1963 mRNAs (1254 upregulated and 709 downregulated) at the transcriptional level were identified as remarkably differentially expressed in the x90 group. A total of 507 DElncRNAs and 2208 DEmRNAs were identified in both the x12A and x90 groups at the gene level. Meanwhile, 124 DElncRNAs and 441 DEmRNAs were identified at the transcriptional level (Figure 5).

### Gene Ontology and Kyoto Encyclopedia of Genes and Genomes Enrichment Analysis

**FIGURE 6:**
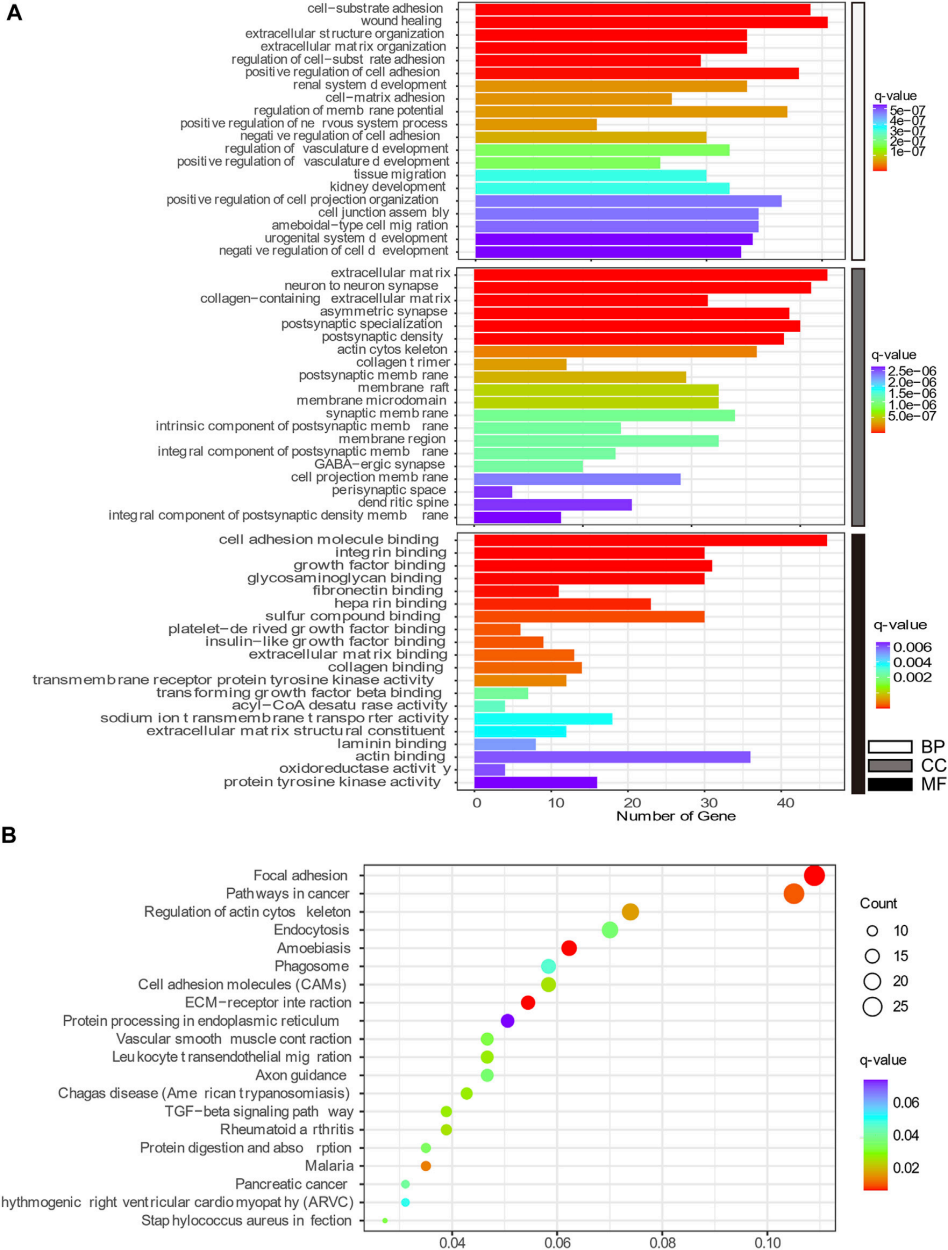
Top 20 GO and KEGG pathway enrichment annotations. **(A)** Biological process (white), cellular component (gray), and molecular function (black). The horizontal axis represents the gene number, which was enriched on the GO term, and the vertical axis shows the GO term name from the analysis of common DEmRNAs shared by both the x12A and x90 groups identified at the gene level. The node color changed gradually from blue to red in ascending order according to the negative log10 (q-value). **(B)** Top 20 KEGG pathway-enriched analyses. The size of the spot indicates the number of enriched genes in the pathway, and the color of the spot indicates the significance level of the enriched pathway. The horizontal axis shows the gene ratio of the GO term, and the vertical axis shows the KEGG term name.

GO enrichment and KEGG pathway analyses were conducted to better understand the mechanisms associated with the action of Aβ and senescence on astrocytes. The highly enriched GO terms in Biological Process (BP), Cellular Components (CC), and Molecular Function (MF) were “wound healing,” “adhesion,” “extracellular matrix,” “neuron to neuron synapse,” “integrin binding,” and “cell adhesion molecule binding” (Figure 6A). Meanwhile, the KEGG pathway analysis of DEGs in x12A astrocytes focused on “focal adhesion,” “regulation of actin cytoskeleton,” “cell adhesion molecules,” “extracellular matrix (ECM)-receptor interaction,” “axon guidance,” “TGF-beta signaling pathway,” and “protein processing in endoplasmic reticulum” (Figure 6B). DEGs in senescent astrocytes were also enriched for similar signaling pathways. The DEmRNAs of both the x12A and x90 groups were enriched in the focal adhesion pathway, ECM-receptor interaction pathway, cell adhesion molecules, axon guidance, and regulation of the actin cytoskeleton signaling pathway. ECM-associated genes Collagen XI alpha 1 (*Col11a1*), platelet-derived growth factor receptor A (*Pdgfra*), Rac Family Small GTPase 2 (*Rac2*), actin beta (*Actb*), Collagen IX alpha 1 (*Col9a1*), Integrin Subunit Alpha 6 (*Itga6*), and Collagen Type I Alpha 1 Chain (*Col1a1*) were enriched in both the focal adhesion pathway and ECM-receptor interaction pathway.

### Protein–Protein Interaction Network Analysis

**FIGURE 7:**
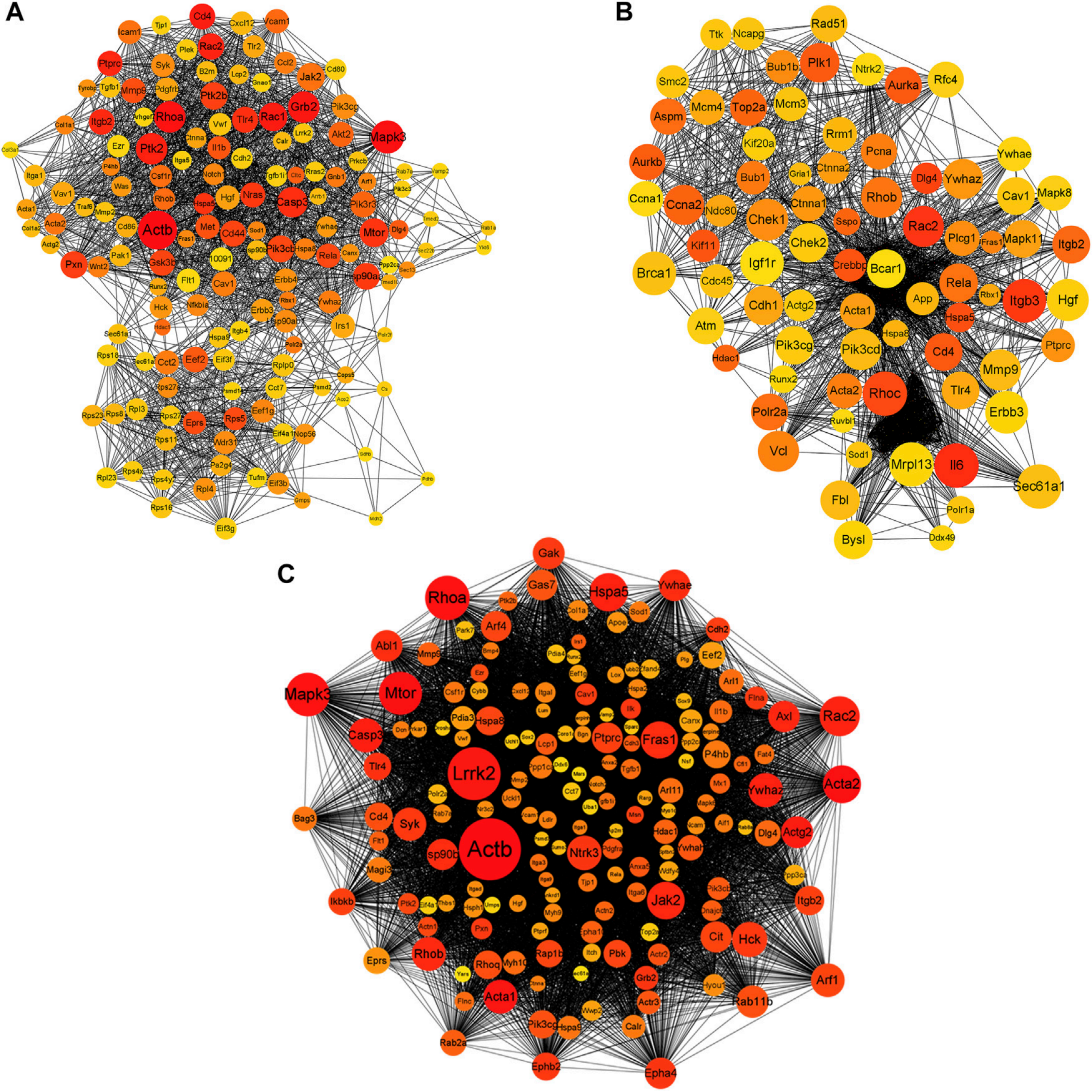
PPI network of differentially expressed genes. **(A)** PPI network of differentially expressed genes in astrocytes treated by Aβ42 oligomers (x12A vs x12). **(B)** PPI network of differentially expressed genes in astrocytes cultured for 90 days *in vitro* (x90 vs x12). **(C)** PPI network of differentially expressed genes in astrocytes treated by Aβ42 oligomers and cultured for 90 days *in vitro* (in both x12A and x90 compared with x12). The size and color of nodes represent their degree and number of interactions, respectively.

To explore the interactions between the proteins expressed by DEGs, PPI networks were constructed using the screened DEGs (the DEmRNAs shared in the x12a vs x12 and x90 vs x12 comparisons identified at the gene level). *Actb*, Mitogen-Activated Protein Kinase 3 (*Mapk3*), Ras homolog family member A (*Rhoa*), Growth Factor Receptor Bound Protein 2 (*Grb2*), *Rac1*, CD4 Molecule, Protein Tyrosine Kinase 2 (*Ptk2*), *Rac2*, Protein Tyrosine Phosphatase Receptor Type C, and Integrin Subunit Beta 2 were considered hub genes in the PPI network of the effect of Aβ_42_ (Figure 7A). Catenin Beta 1, AKT Serine/Threonine Kinase 1, Tumor Protein P53, Ubiquitin A-52 Residue Ribosomal Protein Fusion Product 1, *Actb*, SRC Proto-Oncogene, Non-Receptor Tyrosine Kinase, *Rhoa*, *Mapk3*, Itg Subunit Beta 1, and *Grb2* were identified as crucial genes in the PPI network of senescent astrocytes (Figure 7B). *Actb*, *Mapk3*, *Rhoa*, *Grb2*, *Ptk2*, *Rac2*, *Caspase 3*, Mechanistic Target of Rapamycin Kinase, *Ptk2* Beta, Glutamyl-Prolyl-TRNA Synthetase, Ribosomal Protein S5, Eukaryotic Translation Elongation Factor 2, and Discs Large MAGUK Scaffold Protein 4 were identified among the top 30 hub genes in both Aβ_42_-treated astrocytes and naturally senescent astrocytes (Figure 7C).

### Competitive Endogenous RNA Network of DE mRNAs, Long Non-Coding RNAs, Circular RNAs, and miRNAs

**FIGURE 8:**
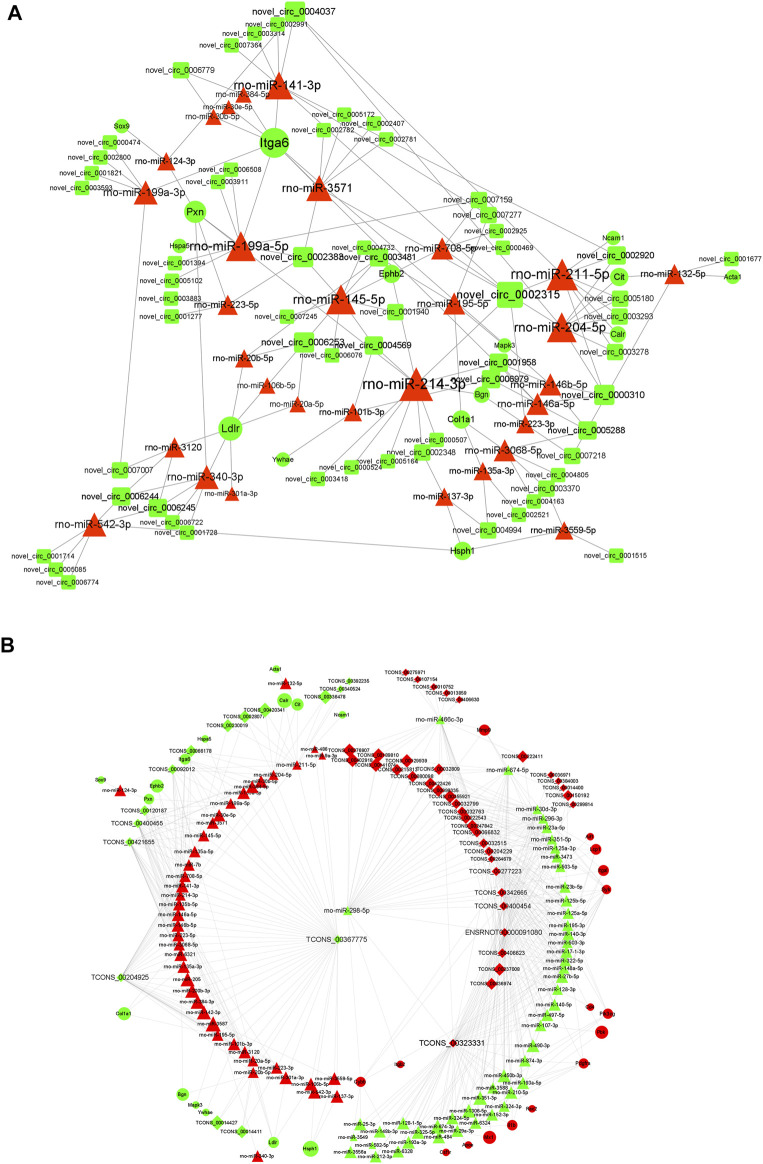
ceRNA interaction network of circRNA and lncRNA. **(A)** Competing endogenous RNA (ceRNA) interaction network of circRNA-miRNA-mRNA. **(B)** Competing endogenous RNA (ceRNA) interaction network of lncRNA-miRNA-mRNA. Red represents upregulated expression, whereas green represents downregulated expression. Round rectangle nodes represent circRNAs, triangle nodes represent miRNAs, and ellipse nodes represent mRNAs. circRNA, circular RNA; lncRNA, long non-coding RNA; miRNA/miR, microRNA; mRNA, messenger RNA; FC, fold change.

The starBase database and miRanda database were used to predict the target miRNAs of lncRNA and circRNAs and the target mRNAs of miRNAs. According to the “ceRNA hypothesis,” lncRNA-miRNA-mRNA and circRNA-miRNA-mRNA ceRNA networks were established (Figures 8A,B) by integrating the expression profiles and regulatory relationships of the lncRNAs, circRNAs, miRNAs, and mRNAs. Notably, rno-miR-199a-5p, rno-miR-145-5p, rno-miR-204-5p, rno-miR-211-5p, and rno-miR-214-3p might play key roles in the circRNA-miRNA-mRNA ceRNA network, regulating the expression of *Itg6*, heat shock protein family H member 1 (*Hsph1*), *Mapk3*, EPH Receptor B2 (*Ephb2*), and *Col1a1*, thus regulating the metabolism and expression of collagen and the ECM (Figure 8A). lncRNAs such as TCONS_00367775, TCONS_00323331, and TCONS_00204925 were identified as potentially regulating the expression of proteins such as *Col1a1*, *Hsph1*, PDZ Binding Kinase, Matrix Metallopeptidase 9, and *Itga6* through multiple miRNAs (Figure 8B). DEGs regulated by the cRNA network were mainly enriched in “macromolecular complex binding,” “protein complex binding,” “collagen binding”, “MAPK signaling pathway,” “Ras signaling pathway,” “Rap1 signaling pathway,” and “cAMP signaling pathway.”

### Validation of Hub Genes in the Alzdata Database

**FIGURE 9:**
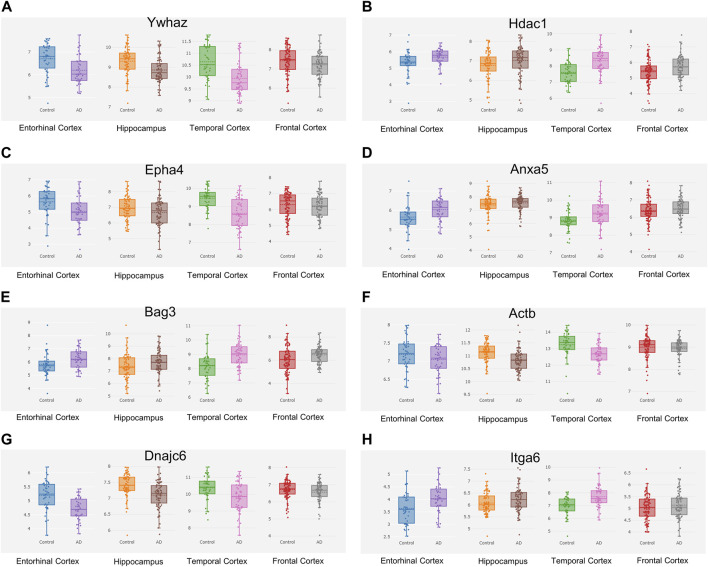
Hub genes screened from the Alzdata database. The hub gene list was submitted to the Alzdata database for cross-checking, and its output showed the variation degree of each gene’s expression in four brain regions, including the entorhinal cortex, hippocampus, temporal cortex, and frontal cortex. The comparison results of the four typical genes in the database are shown in the panel **(A)** *Ywhaz* **(B)** *Hdac1* **(C)** *Epha4* **(D)** *Anxa5* **(E)** *Bag3* **(F)** *Actb* **(G)** *Dnajc6* **(H)** *Itga6*. EC contains 39 controls and 39 AD samples, FC contains 128 controls and 104 AD samples, HP contains 66 controls and 74 AD samples, and TC contains 39 controls and 52 AD samples.

The Alzdata database (http://www.alzdata.org/) is a one-stop database containing the most complete current AD-related high-throughput histology data with high reliability. The screened hub genes were submitted to Alzdata for Normalized_differential and convergent functional genomic rank analysis, and the results showed that genes with altered expression caused by Aβ_42_ and natural aging were similarly documented in the database (Figures 9A–H). These included *ACTB*, *MAPK6, ITGA6*, and *HSPH1*, and some other identified genes had not yet been fully documented. Among the DEGs, seven genes (*YWHAZ*, *HDAC1*, *EPHA4*, *DNAJC6*, *BAG3*, *ANXA5*, and *ACTB*) that were simultaneously differentially expressed in more than two brain regions and had convergent functional genomic scores greater than 3 were screened from the database for subsequent validation which were shown in Table 3.

**TABLE 3:** Genes screened from the Alzdata database.

Gene namacte	CGF score
YWHAZ	5
HDAC1	4
EPHA4	4
ANXA5	4
BAG3	3
ACTB	3
DNAJC6	3

### Validation of Differentially Expressed Genes

**FIGURE 10:**
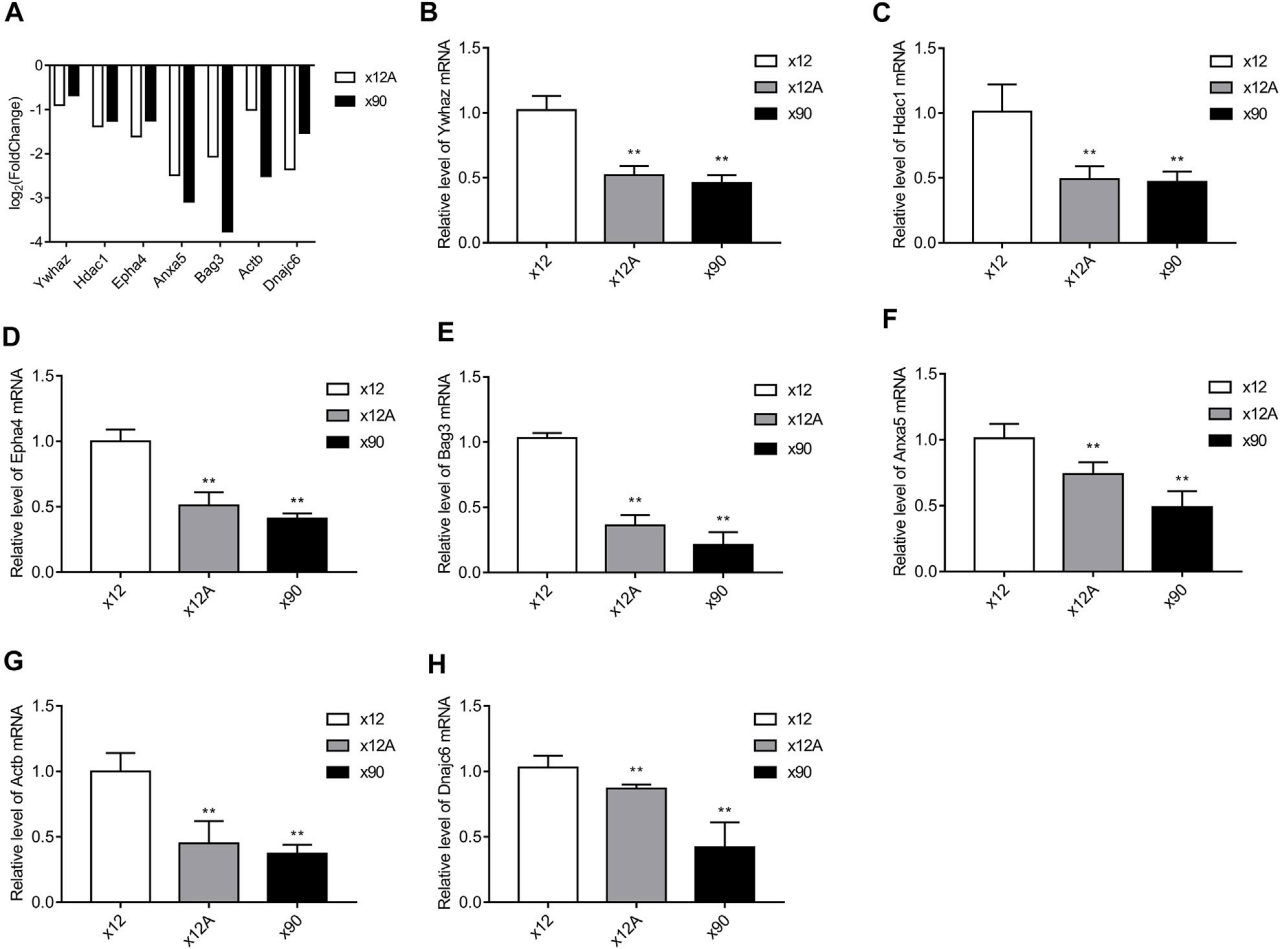
Results of RT-qPCR validation. The hub genes obtained from screening against the Alzdata database were matched against the sequencing results. RT-qPCR was used to detect the expression levels of these genes in Aβ-treated and naturally senescent astrocytes. **(A)** Expression levels of these genes in transcriptome sequencing, expressed as log2 (fold change). Expression levels of these genes in Aβ-treated cells and naturally senescent astrocytes *in vitro* **(B)** *Ywhaz* **(C)** *Hdac1* **(D)** *Epha4* **(E)** *Anxa5* **(F)** *Bag3* **(G)** *Actb* **(H)** *Dnajc6*, as determined by RT-qPCR. *p < 0.05, **p < 0.01, n = 3.

The seven DEGs obtained after the Alzdata database screening were validated by qRT-PCR to confirm whether changes in their expression levels in Aβ_42_-treated astrocytes and naturally senescent astrocytes were consistent with the sequencing data. The changes in expression levels of the seven DEGs in Aβ_42_-treated astrocytes and naturally aged astrocytes showed similar trends (Figure 10), the sequencing data were reliable, and hub genes were available for further investigations.

## Discussion

Our understanding of the role of astrocytes in normal brain functions and disease has deepened over the past few decades. Astrocytes trigger what may be a neuroprotective or detrimental effect in the course of AD (Cai et al., 2017). The disruption of astrocytes and their functions as well as the physiological response of astrocytes to external injury can initiate or aggravate hyperphosphorylated tau (Forny-Germano et al., 2014) and Aβ lesions (Sharma et al., 2012), leading to the formation of amyloid plaques and neurofibrillary tangles as well as neuronal dysfunction. According to the amyloid hypothesis, abnormal accumulation of Aβ in the brain is a major influence driving the pathology of AD. Among all kinds of Aβ isomers, Aβ_40_ and Aβ_42_ were the most important (Qiu et al., 2015). Both Aβ_40_ and Aβ_42_ can aggregate into soluble oligomers through different pathways (Bitan et al., 2003). Aβ_42_ was chosen for our study. The molecular mechanisms underlying the astrocytic response to AD and aging are not yet understood and need to be explored more extensively.

In the present study, *Actb*, *Mapk3*, *Rhoa*, *Grb2*, *Rac1*, CD4 Molecule, *Ptk2*, *Rac2*, Protein Tyrosine Phosphatase Receptor Type C, and Integrin Subunit Beta 2 were identified as crucial genes underlying the influence of Aβ on astrocytes. Catenin Beta 1, AKT Serine/Threonine Kinase 1, Tumor Protein P53, Ubiquitin A-52 Residue Ribosomal Protein Fusion Product 1, Actb, SRC Proto-Oncogene, Non-Receptor Tyrosine Kinase, *Rhoa*, *Mapk3*, Itg Subunit Beta 1, and *Grb2* were identified as key genes underlying the influence of senescence on astrocytes, as determined by topological feature analysis of genes in a PPI network. Subsequently, by establishing a ceRNA network, it was suggested that downregulated lncRNA, such as TCONS_00367775, TCONS_00323331, and TCONS_00204925, might be particularly important for AD and aging; that is, it might act as a ceRNA that downregulates multiple miRNA expression, leading to the downregulation of *Col1a1*, *Hsph1*, PDZ Binding Kinase, Matrix Metallopeptidase 9, and *Itga6*. In addition, downregulated novel-circ-0006245, novel-circ-0006244, novel-circ-0004037, and novel-circ-0002315 might lose the miRNA sponge function to rno-miR-141-3p, rno-miR-3571, rno-miR-199a-5p, rno-miR-211-5p, and rno-miR-145-5p, thus in turn inducing downregulation of the expression of *Itga6*, *Hsph1*, *Mapk3*, *Ephb2*, and *Col1a1.* GO analysis indicated that Aβ and senescence might both be involved in integrin binding, protein binding, extracellular, neuron to neuron synapse, and cell-substrate adhesion functions. KEGG analysis suggested that Aβ and senescence participated in AD pathology by affecting focal adhesion, ECM-receptor interactions, axon guidance, protein processing in the endoplasmic reticulum, the ECM-receptor interaction pathway, and the TGF-beta signaling pathway.

It has been reported that Aβ fibers bind to integrins and mediate Aβ signaling from extracellular sites of Aβ deposition into the cell and ultimately to the nucleus. They do so via the integrin/focal adhesion kinase (FAK)/focal adhesion signaling pathway (Caltagarone et al., 2007). This could induce neuron cell death and activate cell cycle proteins, and activation of the FAK signaling pathway might induce Tau phosphorylation and a decrease in cell adhesion (Caltagarone et al., 2007). Wang et al. (2012) have reported nuclear factor kappa B and FAK activation during Aβ-induced neuronal apoptosis, and *FAK* was found to be upstream of *ERK1/2*, *P38MAPK*, and nuclear factor kappa B. This is in line with findings from Dourlen et al. (2019), who reported that many of the genome-wide association study-defined genes that interact with Tau pathology were involved in the focal adhesion complex, mainly downstream of integrins; the authors also found that members of the focal adhesion pathway might act as modulators of amyloid precursor protein metabolism, which highlights the role of core focal adhesion proteins in AD development. *RTK*, *SRC* Proto-Oncogene, non-receptor tyrosine kinase, *Rac*, and actin were found to be involved in the focal adhesion pathway. Additionally, the focal adhesion pathway played a crucial role in synaptic plasticity (Monje et al., 2012). Therefore, in the case of aggregated Aβ toxicity in both SH-SY5Y neuroblastoma and primary neurons, ECM stiffness helps to regulate cell shape, adhesion, differentiation, survival, and other cellular behaviors (Kruger et al., 2020). Henriques et al. (2015) revealed that Aβ mediated the effects on microtubules and actin networks and stimulated skeleton-related pathological events in AD. In the present study, GO and KEGG analyses showed consistent results, whereby differentially expressed RNAs were enriched in the focal adhesion pathway, ECM receptor interaction pathway, and binding and protein binding terms. As mentioned above, in existing studies, there has been evidence that the effects of Aβ and senescence on neuronal cells are related to signaling pathways such as ECM receptors and focal adhesion. In the present study, we explored the effects of Aβ and senescence on astrocytes, which are closely related to neurons, and showed that Aβ and senescence have significant effects on the expression of proteins related to signaling pathways such as ECM receptors and focal adhesion in astrocytes, which are associated with intercellular communication in different cells. The effects of Aβ and senescence on astrocytes may be affecting neuronal functions through potential intercellular interactions.

*RHO* GTPases are a subfamily of *Ras* superfamily proteins, including more than 20 key proteins that have been identified, with *RHOA*, *RAC1*, and *CDC42* being the classical proteins of the family (Guiler et al., 2021). It has been reported that *RHO* GTPases link multiple intracellular responses with cell surface signals, which regulate actin dynamics for cell migration and cell–cell adhesion, polarization, vesicle trafficking, and the cell cycle (Zegers and Friedl, 2014). Arrazola Sastre et al. (2020) revealed that *RHO* and *RHO* families could switch signaling pathways in neurodegenerative disease. These are involved in almost all stages of brain development (Zamboni et al., 2018). *PTK2* and *FAK* have been demonstrated to be regulators of the *PTK2-TBK1-SQSTM1* axis in *TAR* DNA-binding protein-associated neurodegenerative diseases (Lee et al., 2020). In line with Juliane et al. (Obst et al., 2021), who reported that integrin might be a crucial factor in the regulation of *TREM2* signaling regulation in adhesion and migration functions in AD pathology, we also detected differentially expressed integrin family genes. This indicates that ECM remodeling is essential for normal neuronal and glial development and for establishing adequate synaptic signals. This could explain why ECM dysfunction is frequently seen in neurodegenerative diseases such as AD (Bres and Faissner, 2019). Therefore, as demonstrated by the PPI network results and GO and KEGG analyses, subfamily proteins of the Ras superfamily may link integrin with its focal adhesion functions, and the ECM receptor interaction pathway may be affected by Aβ and senescence of astrocytes, which in turn promotes the pathological progression of AD.

Accumulating evidence has demonstrated that lncRNA-miRNA-mRNA and circRNA-miRNA-mRNA networks may play roles in the pathology of AD (Zhang et al., 2021), including involvement in biological metabolic processes, the cGMP-PKG signaling pathway, the Hippo signaling pathway, and axon guidance. An increasing number of ceRNA networks have been found to be associated with the pathogenesis of AD (Huaying et al., 2020; Tang et al., 2021; Zhang et al., 2021). However, to the best of our knowledge, the ceRNA networks in astrocytes affected by Aβ and senescence have not been revealed. In the present study, rno-miR-141-3p, rno-miR-3571, rno-miR-199a-5p, rno-miR-211-5p, and rno-miR-145-5p were demonstrated to be key miRNAs in the ceRNA network affected by Aβ and senescence. lncRNA TCONS_00367775, TCONS_00323331, and TCONS_00204925 were predicted to regulate the miRNAs, thus suppressing the expression of *ITGA6*, *HSPH1*, *MAPK3*, *EPHB2*, and *COL1A1*. The target gene Itga6 encodes a peptide chain that is one of the subunits of the integrin family and which is closely associated with the ECM receptor signaling pathway; moreover, *ITGA6* has been found to be associated with cell proliferation and cell motility in tumor-related studies (Muir et al., 2020). *HSPH1* has been found to be associated with proteolysis in differentiated neurons (Deane and Brown, 2017). *EPHB2* (Ibrahim et al., 2018; Pan et al., 2020) TNF-α/nuclear factor-kappa B signaling has been reported to worsen cognitive impairment (Tang et al., 2019). *PDGFA* is important for the growth and maintenance of the nervous system (Cardona et al., 2021). The predicted ceRNA networks in the present study showed that Aβ and senescence might affect lncRNA TCONS_00367775, TCONS_00323331, and TCONS_00204925 and multiple circRNAs to sponge rno-miR-141-3p, rno-miR-3571, rno-miR-199a-5p, rno-miR-211-5p, and rno-miR-145-5p to downregulate ECM expression and impact cell proliferation and apoptosis.

In conclusion, the present study identified several DEGs and ceRNA networks that might underlie the influence of Aβ and senescence on astrocytes. Additionally, the molecular mechanisms of the two main pathological factors of AD—Aβ and aging—on astrocyte interactions were highlighted in processes such as ECM and focal adhesion. This indicates that the effects of Aβ and aging on astrocytes at the transcriptome level may play an essential role in the pathogenesis of AD. However, the present work has some limitations. First, the sample size was not large enough, and including more samples, even from patients with AD, would be more valid for the study. Due to the conditions, we could not conduct a more in-depth experimental study to intervene in the crucial genes screened by the transcriptome to explore their specific role in AD pathogenesis. Future experimental investigations will be employed to validate the function of DEGs and the predicted ceRNA network and to further investigate the effects of related genes on oxidative stress, apoptosis, and neuroinflammation in the nervous system. In the present study, we explored the effect of Aβ_42_ on the transcriptome level of astrocytes, and studies on other forms of Aβ such as Aβ_40_ may be conducted in the future.

## Data Availability

The datasets presented in this study can be found in online repositories. The name of the repository and accession number can be found below: SRA, NCBI; PRJNA807497.
